# Evaluation of the redox alteration in Duchenne muscular dystrophy model mice using in vivo DNP-MRI

**DOI:** 10.1038/s44303-024-00058-8

**Published:** 2024-12-05

**Authors:** Hinako Eto, Masaharu Murata, Takahito Kawano, Yoko Tachibana, Abdelazim Elsayed Elhelaly, Yoshifumi Noda, Hiroki Kato, Masayuki Matsuo, Fuminori Hyodo

**Affiliations:** 1 Center for Advanced Medical Open Innovation, Kyushu University, 3-1-1 Maidashi, Higashi-ku, Fukuoka, 812-8582 Japan; 2 Department of Radiology, Gifu University, 1-1 Yanagido, Gifu, 501-1194 Japan; 3 Department of Food Hygiene and Control, Faculty of Veterinary Medicine, Suez Canal University, Ismailia, Egypt; 4 Department of Frontier Science for Imaging, Gifu University, 1-1 Yanagido, Gifu, 501-1194 Japan; 5 Innovation Research Center for Quantum Medicine, Graduate School of Medicine, Gifu University, 1-1 Yanagido, Gifu, 501-1194 Japan; 6 Department of Pharmacology, Graduate School of Medicine, Gifu University, 1-1 Yanagido, Gifu, 501-1194 Japan; 7 Center for One Medicine Innovative Translational Research (COMIT), Institute for Advanced Study, Gifu University, 1-1 Yanagido, Gifu, 501-1194 Japan

**Keywords:** Biological techniques, Biophysics, Diseases

## Abstract

Duchenne muscular dystrophy (DMD) is a genetic muscular disease and is the most common type of muscular dystrophy in Japan. Noninvasive magnetic resonance imaging (MRI) can be used for follow-up evaluation of myositis and muscular dystrophy, including DMD and inflammation is evaluated based on the increased muscle water as evaluated by T2-weighted MR images. However, in MDM, the redox status has not been evaluated non-invasively during the disease progression. We assessed the inflammation via the redox status in experimental animal disease models using in vivo dynamic nuclear polarization MRI (DNP-MRI) with a redox probe. The current study aimed to evaluate the skeletal muscle of mdx mice, a DMD model, in which muscle fiber necrosis, inflammation, and muscle regeneration were chronically repeated. Results showed that the reduction rate of Carbamoyl-PROXYL (CmP), one of the redox probes, radicals in mdx mice increased compared with that in normal mice. In vitro, more mitochondria or macrophages enhanced the radical form decay reaction by reducing CmP. Due to muscle fiber damage, the mdx mice had a lower mitochondrial concentration in the gastrocnemius muscle than the normal mice. However, the in vivo DNP-MRI results strongly reflected the increased reduction of CmP radicals by macrophages. In conclusion, in vivo DNP-MRI, a noninvasive imaging method is useful for locally evaluating skeletal muscle inflammation.

## Introduction

Muscular dystrophy is a generic term for hereditary muscular diseases in which muscle atrophy and weakness gradually progress with repeating destruction/degeneration (muscle necrosis) and regeneration of muscle fibers. It is classified into various disease types according to age of onset, genetic type, and clinical course. Among all hereditary muscular diseases, Duchenne muscular dystrophy (DMD) is the most frequent type and results in early loss of muscle function. DMD is an X-linked neuromuscular disorder caused by a dystrophin gene mutation (locus in Xp21.2), leading to a defect in or absence of the cytoskeletal protein dystrophin in cardiac and skeletal muscle fibers^1,2^. The lack of dystrophin triggers a complex cascade of events, such as increased muscle fiber membrane permeability, calcium influx, reactive oxygen species (ROS) production, and cellular necrosis. Chronic muscle degeneration results in disorganized and persistent accumulation of inflammatory cells, which contribute to disease progression^3^.

DMD is diagnosed based on physical examination findings, family history, and laboratory test results. Molecular diagnostic techniques such as microarray and muscle biopsy can also be used^4^. Histopathologically, the histologic abnormalities in DMD are not commonly specific to this disease. Muscle fiber necrosis, regeneration, and inflammatory cell infiltration are observed. Moreover, the histopathological findings include circularization of the muscle fiber cross-section, increased endogenous nuclei, and expansion of the interstitium^5^.

The most widely used animal model for DMD is the C57BL/10-mdx mouse, which has mutations in the X-linked gene (Dmd) that encodes dystrophin in the sarcolemma. This model presents a DMD skeletal muscle phenotype with age-related decline in strength/power, elevated creatine kinase levels, muscle necrosis, and respiratory issues caused by diaphragm degeneration^6^. Other studies using animal models and humans have revealed that activating pathological cascades (calcium influx, inflammatory immune cell infiltration, cytokines, and proteolytic enzyme activation) can promote DMD progression^7^. In DMD, monitoring local inflammation can help assess disease progression and determine drug therapy’s efficacy.

In terms of imaging diagnosis, magnetic resonance imaging (MRI) is a tool used to assess muscle disease progression. Marty et al. showed that the skeletal muscle inflammation/necrosis and fat infiltration can be assessed by muscle T2 relaxometry and water-fat separation techniques, which utilizes the difference between water and fat T1 and T2 relaxivities^8^. Alic et al. have investigated existing MRI sequences and analysis methods that could be used to identify future needs^9^. This review showed a trend toward using T1- and T2-weighted MRI for the semi-qualitative assessment of structural changes in DMD muscle using different grading scales, with increasing use of T2 map, Dixon, and magnetic resonance spectroscopy.

In the pathophysiological mechanisms of DMD that have been summarized for the purpose of therapeutic strategies and biomarker development, inflammation, mitochondrial dysfunction, and reactive oxygen species dysregulation have been shown as contributing factors^10,11^. These disrupt the balance of the redox state. Therefore, it is useful to comprehensively evaluate the inflammatory and redox status of DMD skeletal muscle, which contains lesions of various temporal phases.

**Fig. 1:**
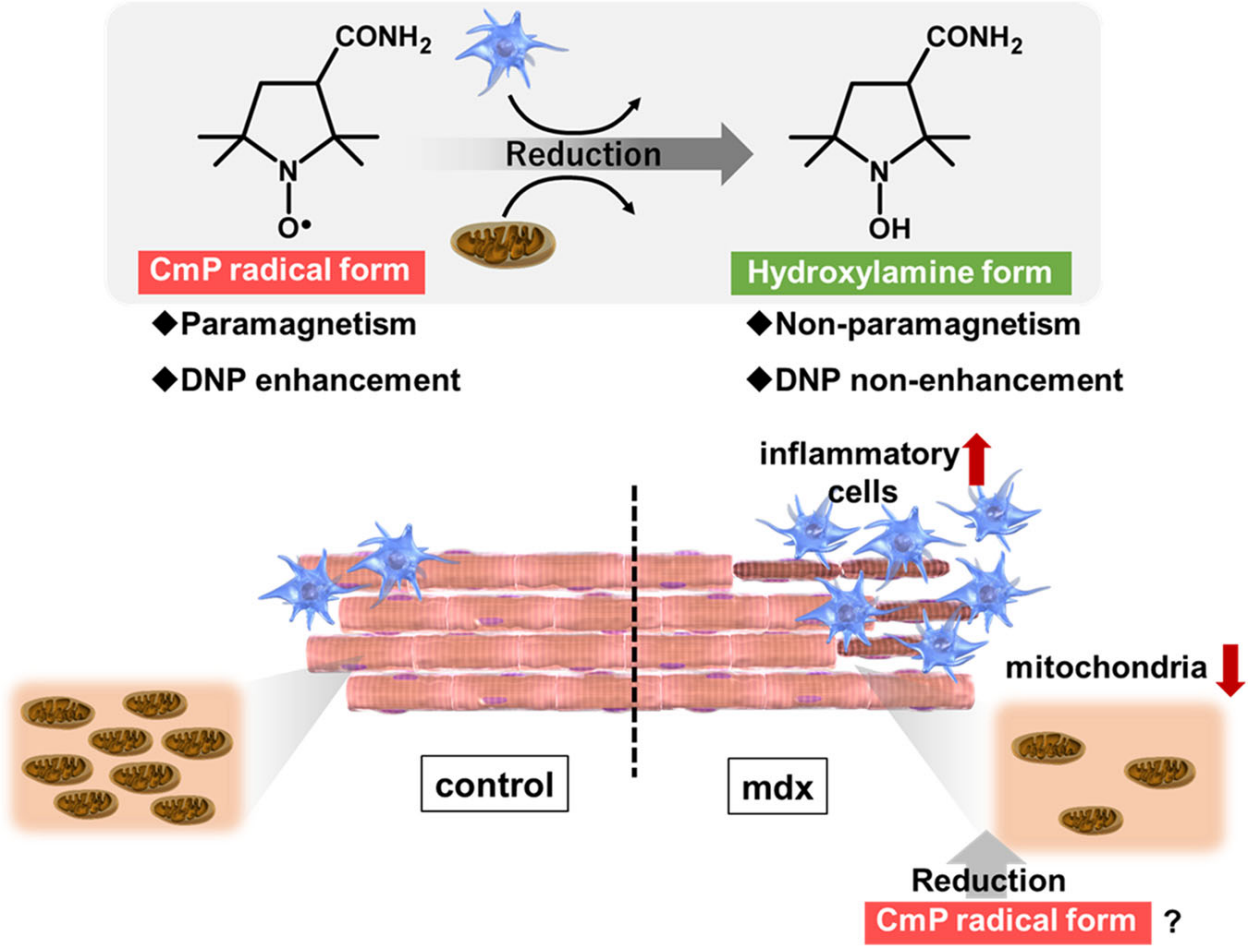
Schematic diagram of the reduction reaction of CmP in skeletal muscle in DMD. Inflammatory cells and mitochondria within myofibers influence the reduction of CmP.

In our previous research, In vivo DNP-MRI, also known as Overhauser-enhanced MRI^12,13^ or proton-electron double resonance imaging^14,15^, irradiates with electron paramagnetic resonance (EPR) at the resonant frequency of the in vivo free radical molecule, induces DNP, and subsequently increases the MRI signal. It can monitor the free radical distribution of nitroxyl compounds administered as probes in vivo based on the interaction between electrons and nuclear spins, known as the Overhauser effect^16^. Several cyclic nitroxyl compounds are stable organic free radicals that have superoxide dismutase (SOD)-like activity^17–19^ and are paramagnetic. The hydroxylamine form, the redox couple of the nitroxyl radical form, is a one-electron reducing reactant and is non-paramagnetic. CmP is a cyclic nitroxyl compound that can be used for biological imaging at noninvasive concentrations and as a redox probe in various biophysical and biochemical experiments^20–22^ (Fig. 1). CmP is highly sensitive in vivo, and the rate of reduction in CmP radicals reflects the redox environment of the tissue^23,24^. Therefore, by monitoring CmP reactions in vivo using in vivo DNP-MRI, changes in the redox state of tissues can be detected with high sensitivity^25–27^. Our previous study showed that in vivo DNP-MRI measurements using CmP can noninvasively detect locally drug-induced acute-phase inflammatory changes in the skeletal muscle^28^. Subsequent studies have revealed that the mitochondria, which are cellular organelles, play an essential role in the reduction reaction of CmP^29^. Therefore, in this study, we aimed to comprehensively assess inflammation, mitochondrial function, and reactive oxygen species status in a mdx mouse model under chronic pathological conditions where inflammation, muscle fiber damage, and muscle fiber regeneration co-exist, and to evaluate the utility of in vivo DNP-MRI measurements for redox imaging (Fig. 1).

## Results

### Redox images of mdx mice at various ages with in vivo DNP-MRI

**Fig. 2:**
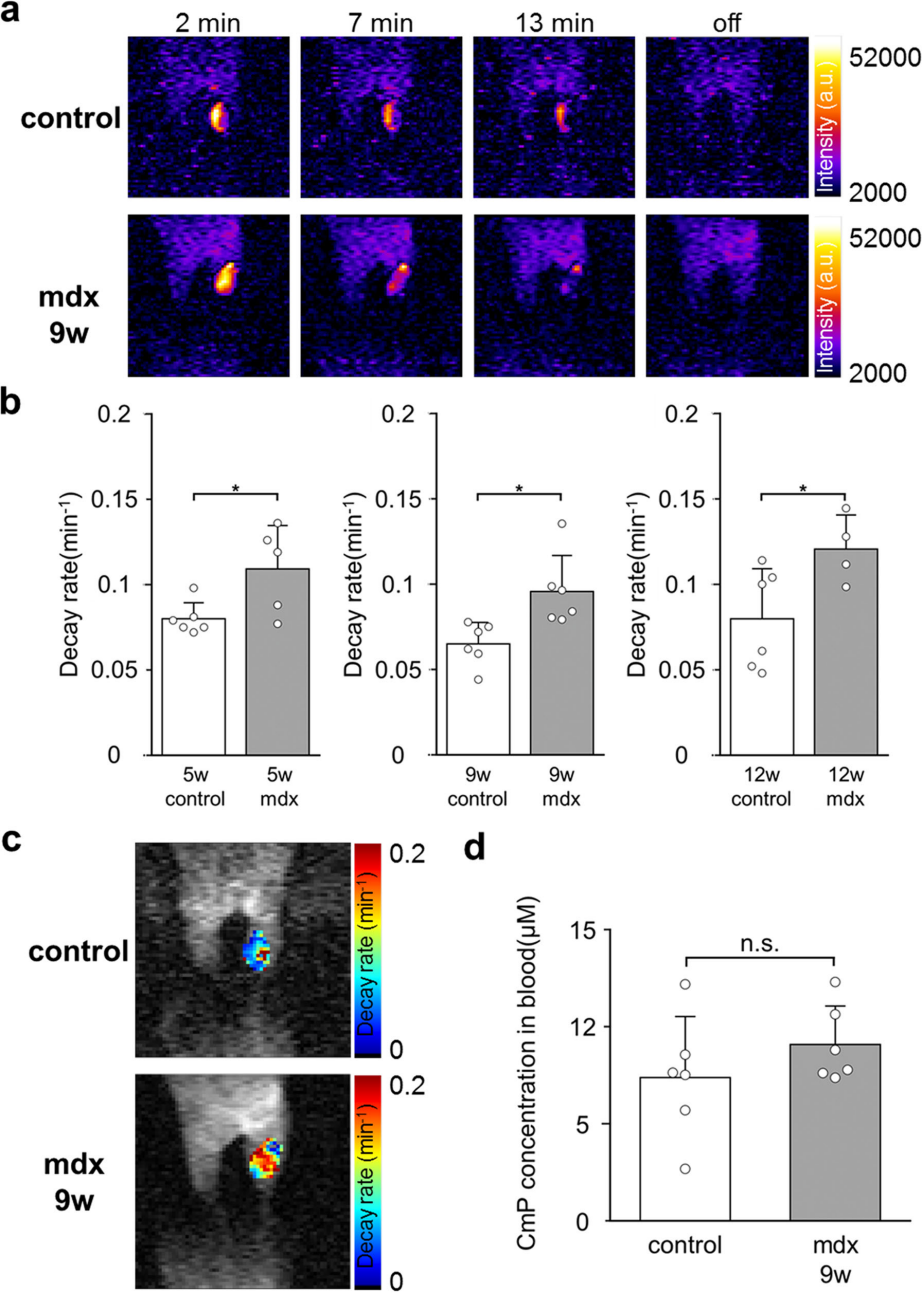
Redox imaging of the gastrocnemius muscle with in vivo DNP-MRI. **a** Representative redox images taken 2, 7, and 13 min after intramuscular administration of the CmP probe, alongside MRI images without EPR irradiation in 9-week-old control mice and 9-week-old mdx mice. **b** Decay rate of CmP radicals in control mice (*n* = 6 per group) and mdx mice at 5-, 9-, and 12-week-old (*n* = 5, 6, and 4), respectively. Data were presented as mean ± SD. *p < 0.05. **c** Redox maps of CmP radicals in control and mdx mice are shown in (**a**). **d** CmP probe leakage into the blood is measured using X-band EPR. Data were presented as mean ± SD (*n* = 6 per group).

To noninvasively assess the redox status of myositis with in vivo DNP-MRI, the gastrocnemius muscle of mdx mice was evaluated using the method described in our previous study^28^. CmP, a cell-permeable nitroxyl radical, was used as a probe to identify extracellular and intracellular compartment redox reactions. In in vivo DNP images, the DNP image intensity disappear when the CmP radical is reduced to the hydroxylamine form. The enhancement of MR image and redox map could be obtained only in the CmP-distributed regions. The image intensity of the CmP radical in the leg was enhanced after 2 min of the intramuscular administration of CmP isotonic solution (2.5 mM, 50 μL). Further, the image intensities of the CmP radical decreased over time in both normal and mdx mice (Fig. 2a). The image intensity of the CmP radical in mdx mice had a faster decay rate than that in normal mice. The decay rate of CmP radicals in the mdx mice group at 5-, 9-, and 12-week-old was evaluated using the Student’s *t* test and compared with that in the control mice group at the same weeks. The average decay rate of CmP radicals in the mdx mice group at 5-, 9-, and 12-week-old (0.109 ± 0.022 min^−1^, *n* = 5; 0.095 ± 0.019 min^−1^, *n* = 6; and 0.12 ± 0.017 min^−1^, *n* = 4) significantly increased compared with that in the control mice group (0.08 ± 0.008 min^−1^, *n* = 6; 0.065 ± 0.011 min^−1^, *n* = 6; and 0.079 ± 0.026 min^−1^, *n* = 6) (*p* = 0.027, 0.012, and 0.042, respectively) (Fig. 2b). The redox map clearly showed differences in decay rates between the control and mdx mice groups (Fig. 2c).

### Evaluation of vascular absorption of CmP probe

Tissue vascular permeability is enhanced by inflammation caused by the constriction of vascular endothelial cells^30^. We validated that the faster decay rate of CmP radicals in mdx mice, measured with in vivo DNP-MRI, was caused by the CmP reduction reaction, not by the absorption of the CmP probe. The blood concentrations of the CmP probe 10 min after intramuscular administration between the control and mdx mice were compared to validate the effect of vascular permeability on the decay of CmP radicals in vivo (Fig. 2d). The blood concentrations of the CmP probe in the control and mdx mice were 7.34 ± 3.51 and 9.07 ± 1.98 µM, respectively. There was no significant difference in the blood CmP probe concentrations between the control and mdx mice (*n* = 6 per group, *p* = 0.288).

### Pathological evaluation of mdx mice

**Fig. 3:**
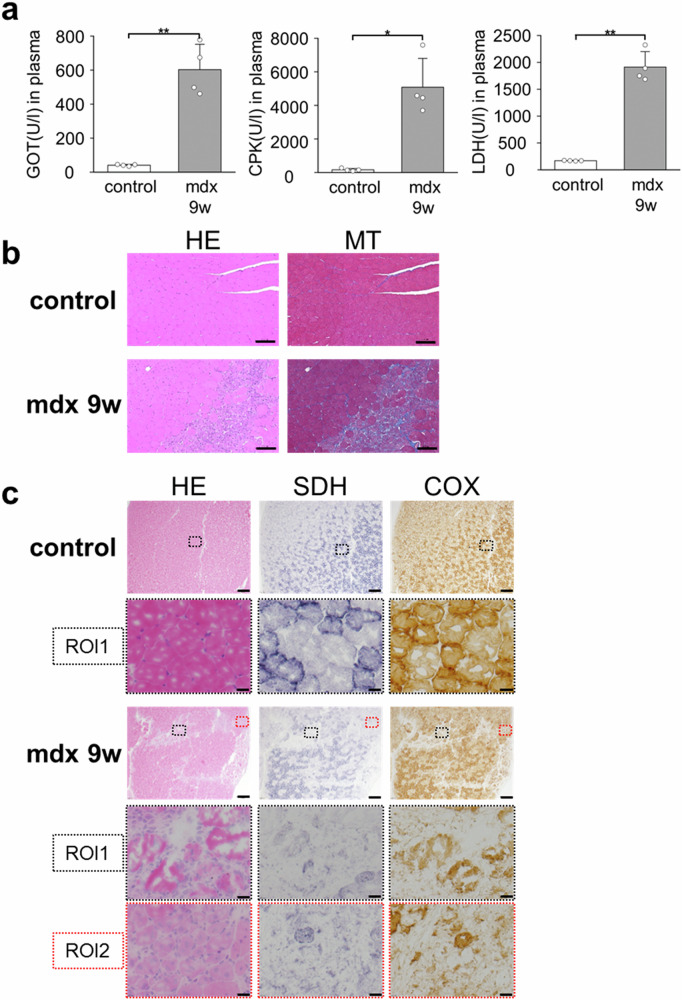
Blood biochemical evaluation and histopathological images of the gastrocnemius muscle in control and mdx mice. **a** Glutamic oxaloacetic transaminase (GOT), creatine phosphokinase (CPK), and lactate dehydrogenase (LDH) plasma concentrations in 9-week-old control and 9-week-old mdx mic. Data were presented as mean ± SD. *n* = 4 per group. *p < 0.05, **p < 0.01. **b** Hematoxylin and eosin (HE) staining and Masson trichrome (MT) staining of paraffin sections of the gastrocnemius muscle in 9-week-old control and 9-week-old mdx mice. Original magnification: ×100. Scale bar represents 100 μm. **c** Hematoxylin and eosin (HE) staining, succinate dehydrogenase (SDH) staining, and cytochrome c oxidase (COX) staining of frozen sections of the gastrocnemius muscle in 9-week-old control and 9-week-old mdx mice, respectively. The upper row images in control mice have the original magnification: ×40. Scale bar represents 200 μm. The lower row images (ROI1: square area surrounded by black dotted lines) had the original magnification: ×400. Scale bar represents 20 μm. In the mdx mice, the upper row images had the original magnification: ×40. Scale bar represents 200 μm. The middle row images (ROI1: square area surrounded by black dotted lines are the defective focus of myofiber cells caused by degenerative necrosis) and the lower row images (ROI2: square area surrounded by red dotted lines are the aggregation of small cells believed to be derived from regenerative cells) had the original magnification: ×400. Scale bar represents 20 μm.

The plasma concentrations of glutamic oxaloacetic transaminase (GOT), creatine phosphokinase (CPK), and lactate dehydrogenase (LDH), which are conventional biochemical markers of muscle injury, were measured. The concentrations of GOT, CPK, and LDH in 9-week-old mdx mice significantly increased compared with those of 9-week-old control mice (Fig. 3a). This finding was similar in mice at any time point (5-, 9-, and 12-week-old) (Fig. S1).

The histopathological findings on hematoxylin and eosin (HE) staining of the gastrocnemius muscle fibers of control mice were as follows: uniform morphology and size, with flattened nuclei on the periphery, tightly packed and converging (Fig. 3b). The gastrocnemius muscle of mdx mice had uniform muscle fibers similar to those of control mice. However, it also had scattered defects of normal muscle fibers caused by focal degenerative necrosis of muscle fibers. Further, in Masson trichrome-stained sections, fibrosis was observed in these focal lesions in mdx mice. Macrophages, neutrophils, and eosinophils were infiltrated in these focal lesions. Further, there was a cluster of small cells with rounded nuclei in the center (Fig. 3c, bottom row, left side image of the mdx mice). These histopathological findings were obtained in mdx mice at 5-, 9-, and 12-week old, and no trends in findings specific to each age were discovered (Fig. S2).

Mitochondrial function significantly affects the radical metabolism of the CmP probe used in in vivo DNP-MRI^29^. Via succinate dehydrogenase (SDH) and cytochrome c oxidase (COX) staining, the histochemical enzymatic activities of the mitochondria in the muscle fibers of control and mdx mice were assessed. In the control mice, the deep layer of the gastrocnemius muscle comprised numerous muscle fibers that were strongly stained with COX and SDH (Fig. 3c). Meanwhile, the superficial layer was composed of COX- and SDH-negative muscle fibers, with COX- and SDH-positive fibers scattered. In the gastrocnemius muscle of the mdx mice, the arrangement of muscle fibers stained with COX and SDH was similar to that in control mice. In addition, the hyalinized and necrotic muscle fibers forming the focal disease in mdx mice tested negative for SDH staining, and it had positive results on COX staining (Fig. 3c, ROI1, middle row center and right side image of mdx mice). Small cells that are regenerated muscle fibers tested negative for COX and SDH staining (Fig. 3c, ROI2, bottom row center and right side image of mdx mice). Hence, muscle fiber necrosis and immature muscle fiber filling reduced mitochondrial content throughout the gastrocnemius muscle.

### Production of ROS in the gastrocnemius muscle of mdx mice

**Fig. 4:**
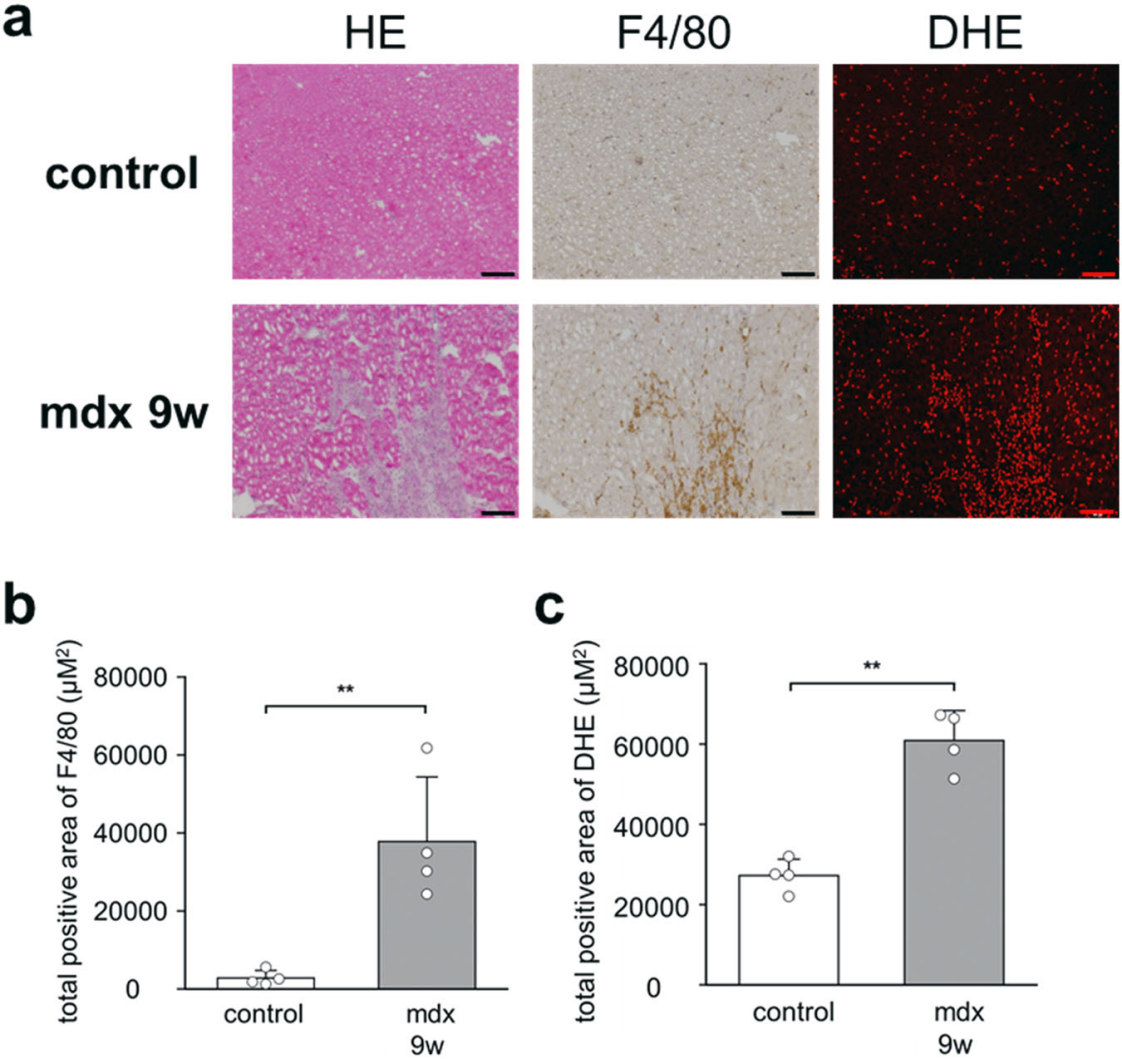
Evaluation of inflammatory cells and ROS in the gastrocnemius muscle in control and mdx mice. **a** Hematoxylin and eosin (HE) staining, immunohistochemistry with anti-mouse F4/80 antibodies, and immunofluorescence with dihydroethidium (DHE) on frozen sections of gastrocnemius muscle in 9-week-old control and 9-week-old mdx mice. The original magnification was ×100. Scale bar represents 100 μm. **b** Total positive area of F4/80 in five fields under microscopy at a magnification of ×200. Data were presented as mean ± SD. *n* = 4 per group. **p < 0.01. **c** Total positive area of DHE in five fields under microscopy at a magnification of ×200. Data were presented as mean ± SD. *n* = 4 per group. **p < 0.01.

The extent of infiltration of mature macrophages, which are the primary inflammatory cells in mdx mice lesions, was evaluated histopathologically from the marker F4/80-positive area (Fig. 4a). In the control mice, positive cells were sparsely scattered in the gastrocnemius muscle. Meanwhile, in mdx mice, numerous F4/80-positive cell infiltrates were found in the focal lesions of muscle fiber necrosis. Further, DHE, a fluorescent dye for detecting ROS generation, is specific to superoxide and hydrogen peroxide. In the control mice, DHE-positive areas were sparsely scattered throughout the gastrocnemius muscle. In contrast, in the mdx mice, high-density DHE-positive areas were observed mainly in areas with many F4/80-positive cells. For F4/80 antibody and DHE dye staining, mdx mice showed a significant increase in positive areas compared with control mice (*n* = 4 per group, *p* = 0.02, 0.0002) (Fig. 4b, c). Hence, large numbers of mature macrophages infiltrated the muscle fiber-deficient foci and increased the production of ROS in the mdx mice.

### CmP radical reduction reaction in mitochondria and macrophages in vitro

**Fig. 5:**
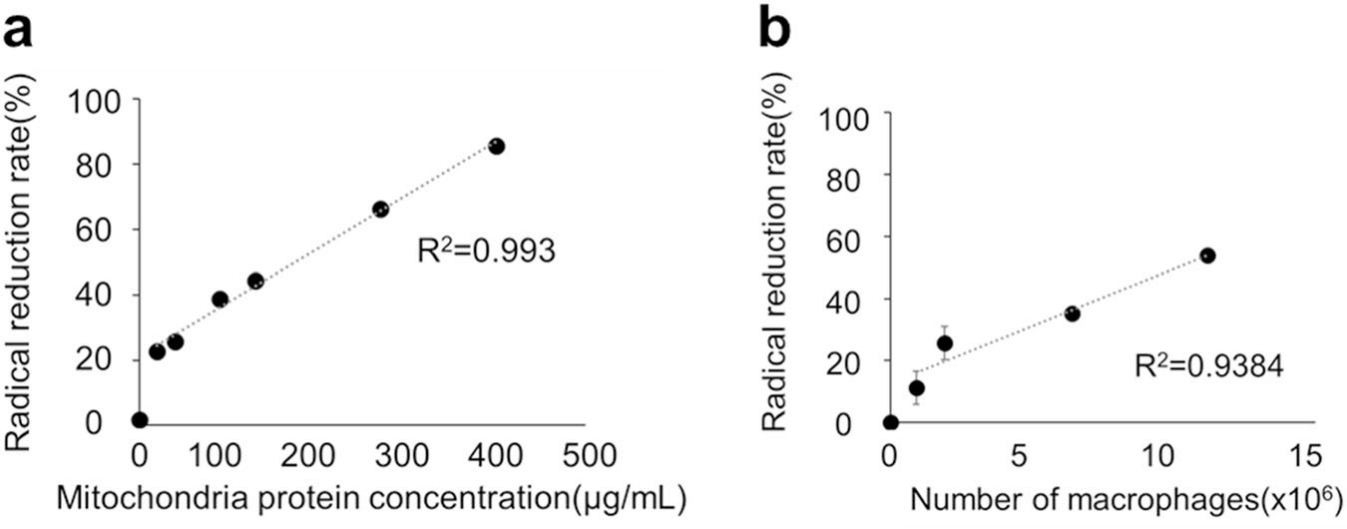
Evaluation of the association between the number of mitochondria or macrophages and the CmP radical reduction rate. **a** Radical reduction rate of CmP due to the reaction with mitochondria. Data were presented as mean ± SD. *n* = 3. **b** Radical reduction rate of CmP due to the reaction with macrophages. Data were presented as mean ± SD. *n* = 3.

The association between the macrophage number or mitochondrial concentration and the CmP radical reducing rate was evaluated. If the mitochondrial concentration was 20–400 µg/mL protein, the radical reduction rate increased linearly with the mitochondrial concentration (Fig. 5a). Further if the macrophage number was 0.9 × 10^6^/mL–6.4 × 10^6^/mL, the radical reduction rate increased linearly with the macrophage number (Fig. 5b). In the mdx mice, the CmP reduction reaction decreases due to a reduced mitochondrial concentration caused by muscle fiber damage. Moreover, the CmP reduction reaction increases due to an elevated ROS caused by macrophages.

## Discussion

We used noninvasive in vivo DNP-MRI imaging to assess the redox status of the gastrocnemius muscle in mdx mice. We evaluated a group of mdx mice at different ages (5-, 9-, and 12-week-olds) and found that the decay rate of CmP radicals was significantly higher than that in normal mice at all ages. These results indicate changes in the redox state of the skeletal muscle in mdx mice^31–37^, which is consistent with myositis skeletal muscle pathology. These also aligns with previous studies showing a significant increase in the decay rate of CmP radicals in a local myositis skeletal muscle compared with in normal skeletal muscle^28^. In a local myositis model, muscle necrosis and inflammatory cell infiltration were induced in the central area of the gastrocnemius muscle where locally administered drugs had infiltrated. Redox alteration in the redox map of DNP-MRI were well corresponded to inflammatory areas by pathological observation. Therefore, it is thought that the enhancement of the decay rate was caused in the areas of inflammatory cell infiltration shown in the histopathological image of mdx mice in Fig. 3b. Consequently, the decay rate of CmP radicals was higher in mdx mice than in control mice composed only of normal muscle cells.

We investigated the absorption of the probe, the level of mitochondrial activity in the gastrocnemius muscle, and the increase in ROS caused by macrophages. These factors increase the decay rate of CmP radicals in mdx mice. There was no difference in the blood concentration between control and mdx mice after a certain period following intramuscular administration of the CmP probe into the gastrocnemius muscle. This led to the fact that the lack of difference in the blood concentration meant that there was no difference in the absorbability of the probe itself. Therefore, we believed that the increased decay rate of CmP in mdx mice was not affected by the absorption of the probe itself.

The results of blood biochemical tests in mdx mice showed a significant increase in the concentrations of biomarkers such as GOT, CPK, and LDH. These elevations suggest that the muscle fibers were injured. Similar results were observed in previous studies using mdx mouse models^38–40^. Elevated levels of these biomarkers can also be used to evaluate patients with DMD. Further evidence of muscle fiber destruction was observed in the histopathological findings. The histological findings of the gastrocnemius muscle in the mdx mice differed from that of the drug induced local myositis model in our previous study. In mdx mice, the pathological state in which muscle fiber damage, inflammation, and muscle fiber regeneration were chronically repeated. Histopathologically, we observed an increase in regenerated muscle fibers in the mdx mice. The small cells with central nucleation were regenerative cells^41–44^. At 3–4 weeks of age, the mdx mice undergo significant muscle fiber degeneration and necrosis, followed by compensatory muscle regeneration that outweighs degeneration^45–47^.

Moreover, 5-, 9-, and 12-week-old mdx mice evaluated via in vivo DNP-MRI presented with muscle fiber damage and regeneration at the same level. Microphotometric assay media for measuring the mitochondrial activity of Complex II (succinate dehydrogenase) and Complex IV (cytochrome c oxidase)^48^ were used to evaluate their potential involvement in CmP radical reduction. The regenerating muscle cells did not show mitochondrial activity on SDH and COX staining. Therefore, the regenerating muscle fibers did not promote the radical decrease in CmP. In addition, the degenerative necrosis of muscle fibers which showed hyaline irregularities on HE staining was negative on SDH staining. A few positive areas were observed on COX staining, but we considered this to be an artifact. The degenerative necrosis of muscle fibers may also not contribute to the radical decrease in CmP.

It has been observed that the infiltration of inflammatory cells, including mature macrophages, in the gastrocnemius muscle of mdx mice led to a significant increase in ROS. This increase in ROS levels enhances the CmP radical reduction reaction. CmP is one of the five-membered ring nitroxyl compounds with an SOD-like effect^49,50^. In the presence of reducing molecules such as reduced nicotinamide adenine dinucleotide (NADH), reduced nicotinamide adenine dinucleotide phosphate, and reduced glutathione (GSH), it converts the nitroxide radical form into the hydroxylamine form via reduction reaction with superoxide^51,52^. Further, the hydroxyl radicals generated from the superoxide via hydrogen peroxide can also be responsible for the response of the radical form to the hydroxylamine form^51,53^. Our previous studies using a mouse model of drug-induced local myositis showed that CmP radical reduction is enhanced by infiltrating inflammatory cells^28^.

We predicted that the reduction of the CmP radical reaction could be caused by muscle fiber damage, which can decrease the number of mitochondria that promote this reaction. However, after conducting an in vivo DNP-MRI evaluation, we found that the CmP radical decay rate in the myositis of mdx mice was significantly faster than that in normal mice. Although there have been reports on the reaction rate of ROS with redox probes such as CmP^54,55^, there have been no reports on the reaction rate with mitochondria. The CmP radical reduction rate increased within a certain period with the increase in the mitochondrial concentration and macrophage count. This study suggests that inflammatory ROS influences the CmP reduction reaction in skeletal muscles affected by myositis rather than by mitochondria.

Recently, a model generated based on a DBA2/J genetic background has also emerged with a high degree of lesion ratio, such as necrosis, regeneration, fibrosis, and inflammation based on a pathological examination of skeletal muscles^40,56^. In vivo DNP-MRI could evaluate the infiltration of inflammatory cells in the skeletal muscle of B10-mdx mice, which did not present with severe muscle damage and dysfunction. The ability of in vivo DNP-MRI to noninvasively detect the local redox state of muscles can be a valuable novel tool for assessing skeletal muscle inflammation. This may contribute to the long-term evaluation of treatment strategies for the early stages of DMD, which often develops in children.

## Methods

### Chemicals

3-Carbamoyl-2,2,5,5-tetramethyl-1-pyrrolidineoxy (carbamoyl-PROXYL), lipopolysaccharides from *Escherichia coli* O111:B4, adenosine 5′-diphosphate sodium salt (ADP), and bovine serum albumin (BSA; lyophilized powder, essentially fatty acid free) were purchased from Sigma-Aldrich Co. (St. Louis, MO, the USA). β-Nicotinamide-adenine dinucleotide, reduced form disodium salt (NADH), glutathione, reduced form (GSH), and disodium succinate were purchased from FUJIFILM Wako Pure Chemical Corporation (Osaka, Japan). Other reagent-grade quality chemicals are commercially available.

### Animals

Female C57BL/10SnSlc (control) mice and female C57BL/10ScSn-Dmdmdx/JicJcl (mdx) mice were purchased from Japan SLC Inc. (Hamamatsu, Japan) and CLEA Japan Inc. (Tokyo, Japan). The mice were 5, 9, and 12 weeks old during the experiments, weighing 13–15, 20–23, and 21–24 g. Intraperitoneal macrophages or liver mitochondria were collected from female Crl: CD1 mice or female C57BL/6N Crl mice and were purchased from Jackson Laboratory Japan, Inc. (Yokohama, Japan). The mice were housed in a climate-controlled room with a 12:12-h light-dark cycle. The mice had free access to water and food (MF diet, Oriental Yeast Co., Tokyo, Japan) and were acclimatized to their environment for 1–3 weeks before the experiments. The Ethics Committee of Animal Experiments, Kyushu University, approved all animal care and experimental procedures. They were conducted following the Guidelines for Animal Experiments of Kyushu University.

### Redox imaging of the gastrocnemius muscle with in vivo DNP-MRI

In vivo redox imaging was performed using a low-field in vivo DNP-MRI system obtained from Japan Redox Inc. (Keller, Fukuoka, Japan). The external magnetic field B_0_ for EPR irradiation and MRI was fixed at 15 mT. The radiofrequency of the EPR irradiation and MRI were 455 MHz and 684 kHz, respectively. This study used a one-turned curved surface coil (longitudinal direction: 20 mm, lateral direction: 32 mm) for EPR irradiation for the skeletal muscle imaging of the hind leg.

DNP-MRI imaging was performed on 5-, 9-, and 12-week-old mdx mice (*n* = 5, 6, and 4, respectively) and normal control mice of the same age (*n* = 6 per group). The mice were anesthetized with 2% isoflurane and held in the prone position, with both hind legs stretched within the sensitive area of the surface coil, which was placed on a special holder. During the procedure, the body temperature of the mice was maintained at 37 °C ± 1 °C with a heating pad. The holder was then placed in the resonator. In vivo DNP-MRI images of the hind legs of a mouse were obtained at 2, 4, 7, 10, and 13 min after the intramuscular administration of carbamoyl-PROXYL (CmP) isotonic solution (2.5 mM, 50 μL, in phosphate-buffered saline [PBS]) to the right gastrocnemius muscles. MRI images were obtained without EPR irradiation. The scanning conditions for the in vivo DNP-MRI experiment were as follows: power of EPR irradiation, 5 W; flip angle, 90°; repetition time × echo time × EPR irradiation time, 1000 × 25 × 500 ms; number of averages, 1; slice thickness (including whole thickness of the mouse), 100 mm; phase-encoding steps, 32; field of view, 40 × 40 mm; and matrix size, 64 × 64 after reconstruction ; scan time, 37 s.

The ImageJ software, available in the public domain^57^, was used for image processing and analysis. In the first image of each mouse, the area of enhanced image intensity by CmP radicals was set as the ROI. The average intensity of each pixel in the ROI was calculated, and the intensity decay rate in the ROI was obtained from the five in vivo DNP-MRI images. The redox map was obtained from the slope of the image intensity of individual pixel in five in vivo DNP-MRI images using a customized Excel macroprogram.

### Measurement of CmP probe concentration in blood

CmP (2.5 mM, 50 μL), an isotonic solution, was intramuscularly administered to the right gastrocnemius muscle of 9-week-old control (*n* = 6) and mdx (*n* = 6) mice. Blood samples were collected 10 min after injection. The CmP probe concentration in the blood was obtained by measuring the total CmP radical concentration of the CmP radical form and the radical form obtained by reoxidizing the hydroxyamine form with potassium ferricyanide using X-band EPR (JEOL Ltd., Tokyo, Japan).

### Plasma biochemistry

Nine-week-old control (*n* = 4) and mdx (*n* = 4) mice were anesthetized with 2% isoflurane. Blood samples (0.5 mL) were collected from the postcaval vein to measure GOT, CPK, and LDH levels. After sacrifice, gastrocnemius muscle samples were also collected from the left and right hind legs for histological assessment, as shown in the section on histological evaluation. The blood samples were immediately centrifuged at 4500 × *g* for 10 min at 4 °C, and the plasma was aliquot and stored at −80 °C for later analysis. GOT, CPK, and LDH plasma concentrations were measured using an autoanalyzer (FUJI DRI-CHEM 4000, Fujifilm Corporation, Tokyo, Japan).

### Histological evaluation

The mice were sacrificed after blood collection, and the left and right gastrocnemius muscles were harvested. The gastrocnemius muscles of control and mdx mice were either fixed in 10% formalin neutral buffer solution for the paraffin section (*n* = 4, 4, respectively) or embedded with OCT compound for the fresh frozen section (*n* = 4, 4, respectively). The paraffin-embedded tissue sections (3 μm; transverse section) were stained with HE. Masson trichrome staining was conducted to evaluate tissue fibrosis (Fig. 3b). The sections were visualized using a high-throughput slide scanner (Axio Scan. Z1; ZEISS, Oberkochen, Germany) at a magnification of ×100.

Fresh frozen sections (5 μm; transverse sections) were stained with HE, succinate dehydrogenase (SDH), and cytochrome c oxidase (COX) (Fig. 3c). SDH and COX staining was performed using the following method. The sections were washed with distilled water three times and incubated with a freshly prepared working solution for 1 h at 37 °C. The SDH working solution was prepared by dissolving disodium succinate (50 mM) and nitro blue tetrazolium chloride (0.5 mM; Wako Chemicals Co., Osaka, Japan) in phosphate buffer (50 mM, pH 7.6). The COX working solution was prepared by dissolving 750 mg of sucrose (Wako Chemicals Co., Osaka, Japan), 5 mg of 3,3′-diaminobenzidine (DAB, DOJINDO LABORATORIES, Kumamoto, Japan), 10 mg of cytochrome c from the bovine heart (Sigma-Aldrich, St. Louis, MO), and 20 µg of bovine liver catalase (Wako, Osaka, Japan) in phosphate buffer (50 mM, pH 7.6). After incubation, sections were rinsed three times with distilled water. SDH-stained sections were mounted with Aqueous (Daido Sangyo, Tokyo, Japan). After dehydration and clearing, COX-stained sections were mounted with Softmount (Wako, Osaka, Japan). The sections were visualized under an inverted fluorescence microscope (IX71 OLYMPUS) at ×40 and ×400 magnification.

### Immunohistochemistry

Nine-week-old control (*n* = 4) and mdx (*n* = 4) mice were sacrificed under 4% isoflurane anesthesia, and the left and right gastrocnemius muscle samples were collected. The gastrocnemius muscles of control and mdx mice were frozen and embedded with OCT compound for fresh frozen sections, respectively. Inflammatory cell infiltration in the gastrocnemius muscles was evaluated in immunostained frozen sections (5 μm; coronal section).

Fresh frozen sections of the gastrocnemius muscles were fixed in acetone at −30 °C. The sections were blocked with 3% normal rabbit serum and then incubated with the primary antibody rat anti-mouse F4/80 (1:500, MCA497GA; Bio-Rad Laboratories Inc., Hercules) at 4 °C overnight. These sections were incubated with the inhibition of endogenous peroxidase activity (peroxidase blocking reagent; Dako Noth America Inc., Carpinteria, CA) and then the secondary antibody biotinylated anti-Rat IgG (H + L) AFFINITYPURIFIED MOUSE ADSORBED (8 ug/mL, BA-4001; Vector Laboratories, Inc., CA, the USA) at room temperature for 30 min. These sections reacted with the avidin-biotinylated enzyme complex using the VECTASTAIN ABC Kit (Vector Laboratories, Inc., CA, the USA). The color was then developed using a peroxidase substrate kit (ImmPACT DAB substrate kit; Vector Laboratories, Inc., CA, the USA). The nuclei were stained with hematoxylin. Finally, the sections were mounted on VectaMount® Permanent Mounting Medium (H-5000 Vector Laboratories, Inc., CA, the USA). The sections were visualized under an inverted fluorescence microscope (IX71 OLYMPUS) at a magnification of ×200 and measured as the total F4/80-positive areas in five fields using the ImageJ software.

### Evaluation of superoxide production using immunofluorescence

The production of superoxide (O2.-.) was evaluated by fluorescent dye staining with dihydroethidium (DHE, Sigma-Aldrich, St. Louis, MO, the USA). Fresh frozen sections (5 μm; coronal section) of the gastrocnemius muscles were washed with PBS three times and incubated with DHE (50 mM) in PBS at 37 °C for 30 min in a humidified chamber protected from light. After mounting with ProLong™ Diamond Antifade Mountant (Thermo Fisher Scientific Inc., MA, the USA), the sections were visualized under a fluorescence microscope (IX71 OLYMPUS) at a magnification of ×100 magnification and were measured as the total fluorescence area in five fields using the ImageJ software.

### Monitoring the redox reaction of macrophages and CmP with X-band EPR

Mouse intraperitoneal macrophages were collected from eight Crl:CD1 mice via intraperitoneal lavage after stimulation with 4% thioglycollate medium (1.5 mL; Nissui Pharma Medical Sales Co., Ltd., Tokyo, Japan) for 72 h. The intraperitoneal lavage solution was lysed with lysis buffer (Red Blood Cell Lysing Buffer Hybri-Max; Sigma-Aldrich St. Louis, MO, the USA) and washed three times with ice-cold Dulbecco’s Phosphate Buffered Saline (D-PBS, without calcium and magnesium). The cell sediment was resuspended in 5 mL of Dulbecco’s Modified Eagle Medium and incubated in a petri dish at 37 °C for 1 h in a CO_2_ incubator. The petri dish was rinsed twice with D-PBS, and the remaining adherent cells were used as macrophage cells. Macrophages in D-MEM were reacted with CmP (50 µM), NADH (5 mM), GSH (5 mM), and lipopolysaccharides (100 ng/mL) in 96 wells. The radical reaction rate was calculated by measuring the CmP radical concentration of the sample immediately after the reaction and the sample after 2 h of incubation at 37 °C under 5% CO_2_ using X-band EPR.

### Monitoring the redox reaction of the liver mitochondria and CmP with X-band EPR

Three C57BL/6N Crl mice that had fasted for 12 h were sacrificed under 4% isoflurane anesthesia, and their liver was resected. The liver mitochondria were isolated by modifying the method used in a previous study. Briefly, the liver homogenized in ice-cold MAS buffer A (220 mM D-mannitol, 70 mM sucrose, 2 mM HEPES, 0.2% BSA, 1 mM EGTA, and 10 mM KH_2_PO_4_, pH 7.2) was centrifuged at 600 × *g* for 10 min to remove nuclei and cell debris. The supernatant was centrifuged at 7000 × *g* for 10 min. The pellet containing the mitochondria was washed twice and finally resuspended with a small volume of MAS buffer B (220 mM D-mannitol, 70 mM sucrose, 2 mM HEPES, 10 mM MgCl_2_, 10 mM KH_2_PO_4_, and 1 mM EGTA, pH 7.2). Then, it was resuspended to 20 mg of protein/mL as the mitochondrial fraction. The mitochondrial fraction suspensions were reacted with CmP (50 µM), NADH (5 mM), GSH (5 mM), disodium succinate (10 mM), and ADP (1.5 mM). The radical reaction rate was calculated by measuring the CmP radical concentration of the sample immediately after the reaction and the sample after 2 h of incubation at 37 °C under 5% CO_2_ using X-band EPR.

### Statistical analysis

Data were presented as means ± standard deviations. Further, data were evaluated using the *f*-test. A *p* value of <0.05 indicated a statistically significant difference and was treated as two samples with equal variance. Meanwhile, a *p* value of ≥0.05 did not show significant differences and was treated as two samples with unequal variance. In the levels of GOT, CPK, and LDH in plasma and the verification of the total F4/80-positive area, the data were evaluated with the Student’s *t* test as two samples with unequal variance on a two-tailed distribution. In the other experiments, the data were assessed using the Student’s *t* test as two samples with equal variance on a two-tailed distribution. A *p* value of <0.05 or <0.01 indicated a statistically significant difference.

## Supplementary information

Supplementary information
