# Supplementary material for: Evaluation of the redox alteration in Duchenne muscular dystrophy model mice using in vivo DNP-MRI
Source: Npj Imaging. 2024 Dec 5;2:52. doi: 10.1038/s44303-024-00058-8 (PMC12118763; doi:10.1038/s44303-024-00058-8)
Supplement: Supplementary file 1 — Supplementary information [file 44303_2024_58_MOESM1_ESM.pdf]

**Supplementary Figure 1**

**Representative blood biochemical evaluation in control and mdx mice.**

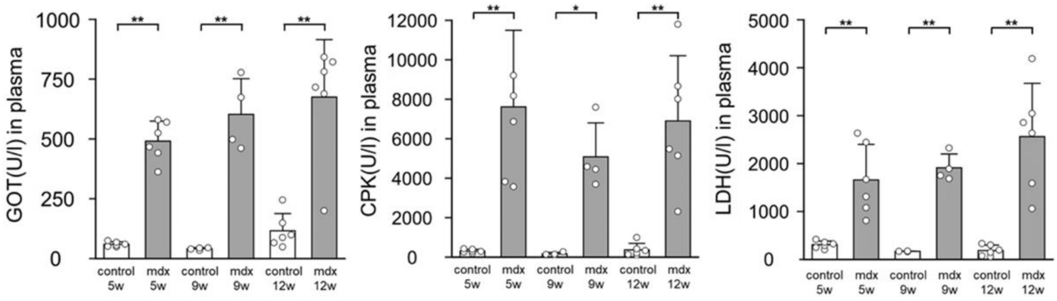


Glutamic oxaloacetic transaminase (GOT), creatine phosphokinase (CPK), and lactate dehydrogenase (LDH) plasma concentrations in normal control mice (n = 6, 4, and 6 ) and mdx mice at 5-, 9-, and 12-week old (n = 6, 4, and 6) Data were presented as mean ± SD. ** $p < 0.01$.

**Supplementary Figure 2**

**Representative histopathological images of the gastrocnemius muscle in control and mdx mice.**

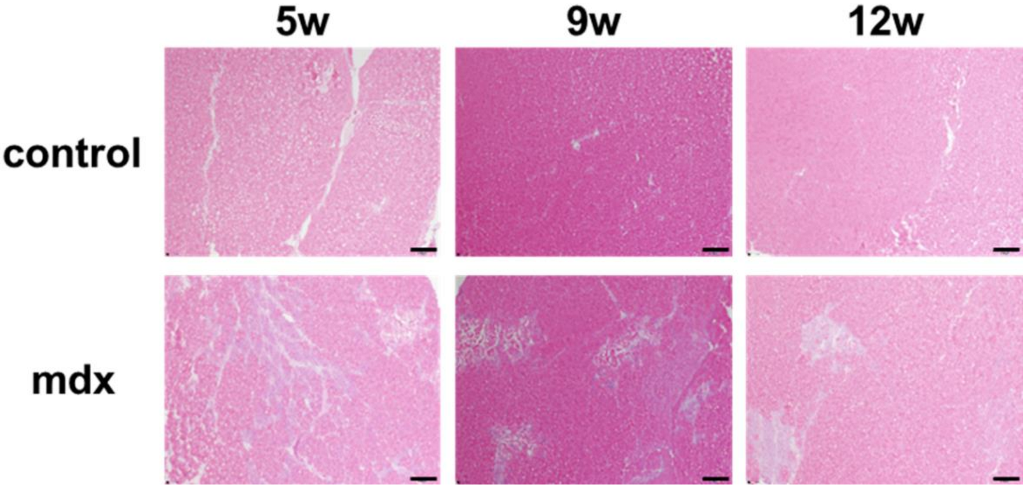


Hematoxylin and eosin (HE) staining of frozen sections of the gastrocnemius muscle in control and mdx mice at 5-, 9-, and 12-week old. Original magnification: × 40. Scale bar represents 200 μm.
